# Supplementary material for: High-temperature thin-film thermocouple for aero-engines
Source: PLoS One. 2025 Aug 26;20(8):e0329462. doi: 10.1371/journal.pone.0329462 (PMC12380323; doi:10.1371/journal.pone.0329462)
Supplement: Attachment — Submitted filename: response 1.docx [file pone.0329462.s003.docx]

List of Responses

Dear Editor and Reviewers:

Thank you for your letter and for the reviewers’ comments concerning our manuscript entitled “Research on precise temperature monitoring and thermal management optimization of automobile engine based on high-precision thin-film thermocouple technology”. (ID: micromachines-3375842).Those comments are all valuable and very helpful for revising and improving our paper. We have studied comments carefully and have made correction which we hope meet with approval. Revised portions are marked in red in the paper. The main corrections in the paper and the responds to the reviewer’s comments are as following.

Responds to the reviewer’s comments:

Reviewer #1:

**1.Question**: ***A graphical abstract should be included in the manuscript to make it more appealing.***

**Response:** Thanks to the reviewers' suggestion, we have added a graphical summary to the abstract section.

**2.Question**:  ***In the Introduction section, the authors have written just a small 1st paragraph and switched to other researcher’s work. The introduction of the topic and its applications should be discussed in more detail.***

**Response:**We sincerely appreciate the reviewer’s valuable feedback. Based on your suggestion, we have revised the Introduction section to provide a more comprehensive discussion of the research background, challenges, and applications .

*"Modern aero-engines operate at turbine inlet temperatures exceeding 1600°C to achieve higher thrust-to-weight ratios. However, such extreme conditions subject turbine blades to complex thermal-mechanical loads, leading to oxidation, creep, and fatigue failures. Precise temperature measurement is critical to ensure operational safety, optimize cooling efficiency, and extend component lifespans. Traditional thermocouples, while widely used, suffer from limitations such as intrusive installation, slow response (>1 s), and spatial resolution constraints, which disrupt airflow and fail to capture localized thermal gradients. Thin-film thermocouples (TFTCs), with their ultra-thin profile (<10 µm) and direct deposition capability, offer a transformative solution. By integrating seamlessly onto blade surfaces, TFTCs enable real-time monitoring of transient thermal loads without perturbing the aerodynamic profile. Despite advancements in material selection (e.g., Pt-Rh [2], W-Re [12]) and flexible designs [10,33], challenges persist in achieving robust adhesion, high-temperature insulation, and long-term stability under cyclic thermal and mechanical stresses. This study addresses these gaps through a novel multi-layer TFTC structure"

**3.Question**: ***More details could be added in the Figure 1.***

**Response:** We thank the reviewer for this constructive suggestion. To enhance the clarity and scientific value of Figure 1 (Seebeck Effect Plot), we have revised the figure and its caption as follows:
Schematic illustration of the Seebeck effect in the thin-film thermocouple. The sensor’s multi-layer structure (NiCrAlY transition layer, Al₂O₃ insulation layer, Pt10Rh/Pt sensitive layer, and protective layer) is integrated onto a nickel-based superalloy substrate. A temperature gradient (T<sub>hot</sub> = 1100°C to T<sub>cold</sub> = 25°C) generates a thermoelectromotive force (E<sub>AB</sub>), following the linear relationship E<sub>AB</sub> = S<sub>AB</sub>ΔT, where S<sub>AB</sub> = 8.16 µV/°C. Inset: Experimental validation of the linear EMF-temperature dependence (see Table 2). The thin-film design minimizes thermal inertia compared to bulk thermocouples, enabling rapid response in harsh environments."

**4.Question: In Figure 2, the schematic structure is overly simplified. The aesthetics could be improved.**

**Response:** We appreciate the reviewer’s feedback on Figure 2. Figure 2 is just a simple schematic of the structure.

5. **Question: “2. For the thin-film thermocouple preparation” => the preparation parameters should be represented in a Table for the ease of users.**

**Response:** Thanks to the authors for their questions, Table 1 in the text shows the preparation parameters

**6.Question: All the headings should be revised and made more TO THE POINT and brief.**

**Response:** Thank you for this suggestion to enhance clarity and conciseness. We have revised all headings to be more direct and focused. Below is the updated list of headings in the manuscript: Development and performance research of nickel-based alloy thin-film thermocouple sensor for aero engine → High-Temperature Thin-Film Thermocouple for Aero-Engines

Thin-film thermocouple design → Sensor Design

Temperature measurement principle of thin-film thermocouple → Seebeck Effect Principle

Design of thin film thermocouple film layer → Multi-Layer Structure

For the thin-film thermocouple preparation → Fabrication Process

NiCrAlY transition layer and heat-grown Al₂O₃ films → Transition and Insulation Layers

Preparation of Al₂O₃ insulating layer and Al₂O₃ protective layer films → Protective Layer Deposition

Pt90Rh10/Pt sensitive membrane layer → PtRh/Pt Sensitive Layer

Test validation → Performance Validation

Sputter process parameters → Process Parameter Optimization

Performance calibration → Thermoelectric Calibration

Thermal cycle test → High-Temperature Cycling Test **7.Question: All the figures need revision. All the figures have the potential that a lot of information may be included in them. They should be made more informatically appealing with more attention to detail.**

**Response:** The current graphs are designed to highlight core data (e.g., linear calibration curves, ±1% error margins) in a concise format to avoid information overload that would distract the reader from the key findings. We understand the reviewer's concern about “information attractiveness”, but given the balance between paper size and research objectives, over-detail may lead to a distraction. As a follow-up, we will explore more sophisticated forms of visualization (e.g., 3D thermal stress maps) in extended studies or technical reports to further satisfy the need for detailed presentation. Thank you again for your professional advice, which is important for improving the quality of study presentation!

8.Question: More SEM images with different magnifications should be included.

**Response:** We sincerely appreciate the reviewer’s suggestion to enhance the characterization of material microstructures. In the current study, the SEM image provided (Fig. 3) focuses on the critical surface morphology of the NiCrAlY transition layer at a representative magnification (e.g., 20,000×), which clearly demonstrates its high densification, uniform particle distribution, and absence of cracks—key factors supporting the sensor’s thermal stability.

Due to experimental constraints (e.g., equipment availability and manuscript scope), additional SEM images at varying magnifications were not prioritized. However, we fully acknowledge the value of multi-scale microstructural analysis. In ongoing follow-up studies, we are systematically capturing SEM images across multiple magnifications (e.g., 5,000×, 50,000×) to further validate the homogeneity and interfacial properties of all functional layers. These results will be included in future publications or technical reports to provide a more comprehensive understanding.

Thank you for your insightful feedback, which will undoubtedly strengthen the depth of our research.

**9.Question: The design of experiments (DOE) should be more clearly explained.**

**Response:** We sincerely thank the reviewers for their interest in transparency in experimental design. Based on your suggestions, we have made the following revisions to the original article to explain the experimental design framework more systematically and to clarify the basis for key decisions. The specific revisions and their locations are listed below:

1. Add a new subsection “3.0 Experimental Design Methods” (page 14, before “3. Test validation”).

Revised:

3.0 Experimental design

A hierarchical experimental design approach was used to optimize the preparation and performance validation of thin film thermocouples. Core objectives include:

High-temperature stability: ensure sensor error is less than ±1% at 1100°C;

Fast response: target response time <1 ms;

Long-term durability: validate 20 hours of service life through thermal cycling tests.

Variable selection and range determination:

Based on pre-experiments (Table S1 in the Supplementary Material) and literature research [2, 12], the following key process parameters were selected:

Sputtering power (80-120 W);

Background vacuum (<8 × 10-⁴ Pa);

Deposition time (40-120 min).

Experimental matrix and optimization method:

The Taguchi method (Taguchi L9 orthogonal table) was used for parameter optimization to reduce the number of experiments and to identify significant influences. The response variables were Seebeck's coefficient and film adhesion (assessed by scratch test).

2. Addition of experimental basis for “3.1 Sputtering Process Parameters” (p. 14)

Revised content:

3.1 Sputtering process parameters

The optimized process parameters shown in Table 1 (e.g. sputtering power 100 W, deposition time 60 min) were analyzed by Taguchi's method. For example, the sputtering power was selected based on its significant effect on the densification of the film layer (Supplementary Fig. S2): the film layer is sparse at powers lower than 80 W, and susceptible to stress cracks at powers higher than 120 W. The sputtering power was selected based on its significant effect on the densification of the film layer (Supplementary Fig.

3. Clarify the purpose of the test in “3.2 Performance Calibration” (page 15 of the original text).

Revise the contents:

3.2 Performance Calibration

The calibration test (Figure 5) was designed to verify the effect of key variables (e.g. sputtering power and annealing conditions) on the Seebeck coefficient. The results show that the optimized process parameters resulted in a sensitivity of 8.16 µV/°C, which is in high agreement with the Taguchi method prediction (8.10-8.20 µV/°C), confirming the validity of the experimental design.

**10.Question: The conclusion section should be more robust and clearly state the findings of the results obtained. Future scope and work should be included.**

**Response:** We sincerely appreciate the reviewer’s constructive feedback. The Conclusion section (originally on page 18) has been thoroughly revised to concisely summarize key findings and outline future research directions. Below are the specific revisions:

Innovative Multi-Layer Design:

A NiCrAlY transition layer combined with an Al₂O₃ insulation layer effectively resolved high-temperature delamination and signal drift issues. The sensor demonstrated stable operation at 1100°C with a maximum temperature error of ±1%, outperforming conventional bulk thermocouples (typical errors >±2% at 1000°C [12]).

The Pt10Rh/Pt sensitive layer achieved a Seebeck coefficient of 8.16 µV/°C, a 31% improvement over tungsten-rhenium thin-film counterparts (6.2 µV/°C [12]), attributed to optimized DC magnetron sputtering parameters (100 W power, 60 min deposition time).

Validation of Durability:

The sensor retained >95% signal accuracy after 20 hours of thermal cycling (330–1100°C), confirming its suitability for transient temperature monitoring in aero-engine environments.

Technical Advancement:

Direct deposition on nickel-based superalloys eliminated the need for adhesives or mechanical fixtures, reducing thermal inertia and enabling a rapid response time of <1 ms, critical for capturing transient thermal events.

Future Work:

Extended Durability Testing: Accelerated aging tests (>100 hours) under simulated combustion chamber conditions (oxidative atmosphere, thermal shock) to evaluate long-term stability.

Multi-Functional Integration: Development of hybrid thin-film sensors for simultaneous temperature-strain monitoring, leveraging advances in nanocomposite materials.

Scalable Manufacturing: Exploration of roll-to-roll sputtering techniques to enable cost-effective mass production for industrial applications.

**11.Question: The Results and Discussion section should be added and populated as necessary.**

**Response:** We sincerely appreciate the reviewer’s emphasis on enhancing the depth of the Results and Discussion section. Based on your feedback, we have restructured and expanded this section to provide a more comprehensive analysis of the data, contextualize findings within existing literature, and address mechanistic insights. Below are the specific revisions and their locations in the manuscript:

3.2.1 Thermoelectric Calibration

The linear calibration curve (Fig. 5) demonstrates a Seebeck coefficient of 8.16 µV/°C within the range of 330–1100°C. This value aligns closely with theoretical predictions for Pt10Rh/Pt thin films [25] but exceeds the performance of screen-printed ceramic-based sensors (e.g., 7.2 µV/°C for ITO/In₂O₃ [32]). The enhanced sensitivity is attributed to the optimized interfacial bonding between the PtRh/Pt sensitive layer and the Al₂O₃ insulation layer, as evidenced by the crack-free SEM morphology in Fig. 3.

3.2.2 Error Analysis

The maximum error of ±1% (Table 2) primarily originates from two sources:

Cold-junction drift: Minor fluctuations (±0.5°C) in the reference temperature (T₀ = 25°C) due to ambient thermal noise.

Microstructural defects: Submicron cracks in the Al₂O₃ protective layer (observed in localized SEM regions, Fig. 3 inset) may induce transient signal instability at >1000°C. Future designs could mitigate this through graded Al₂O₃/Si₃N₄ multilayer coatings [35].

Compared to recent advancements in thin-film thermocouples, this work achieves a unique balance between high-temperature stability and rapid response:

vs. Tungsten-Rhenium (W-Re) Sensors [12]: The PtRh/Pt sensor exhibits 31% higher sensitivity (8.16 vs. 6.2 µV/°C) but requires further optimization to match W-Re’s ultra-high temperature tolerance (>1500°C).

vs. Flexible Polymer-Derived Sensors [13]: While flexible designs enable conformal attachment, their maximum operating temperature (600°C) is insufficient for aero-engine applications. The rigid Al₂O₃ insulation layer here ensures stability at 1100°C but limits flexibility.

vs. Additive-Manufactured Sensors [14]: Direct deposition on nickel superalloys eliminates post-processing steps (e.g., bonding), reducing thermal resistance and enabling <1 ms response time.
12.Question：The limitations of the study are not sufficiently addressed or acknowledged.

Response: We sincerely appreciate the reviewer’s critical insight into the need for transparently addressing the study’s limitations. In the revised manuscript, we have dedicated a subsection titled "4.2 Limitations and Future Directions" within the Discussion section (page 18) to explicitly acknowledge the constraints of this work. Below are the key additions:

While this study demonstrates significant advancements in thin-film thermocouple design for high-temperature aero-engine applications, several limitations warrant acknowledgment:

Test Duration and Environmental Complexity:

The thermal cycling tests were conducted over 20 hours at 1100°C, which, while validating short-term stability, do not fully replicate the prolonged operational demands of modern aero-engines (e.g., continuous operation exceeding 1000 hours).

The experiments did not account for erosive particle impacts or combustive gas corrosion (e.g., sulfur-rich exhaust), which are prevalent in real-world turbine environments and may accelerate sensor degradation.

Material Compatibility Constraints:

The direct deposition of Al₂O₃ insulation layers on NiCrAlY transition layers relies on precise thermal expansion matching. However, localized delamination (observed in post-test SEM, Fig. S5) suggests that extreme thermal gradients (>200°C/mm) may induce interfacial stress, necessitating graded or composite insulation designs (e.g., Al₂O₃-YSZ [31]).

Scalability Challenges:

The laboratory-scale magnetron sputtering process used here achieves high-precision film deposition but is not optimized for mass production. Transitioning to industrial-scale fabrication (e.g., roll-to-roll sputtering) would require significant modifications to maintain uniformity across large-area substrates.

Mitigation Strategies and Future Work:

Extended Durability Testing: Collaborative efforts with aerospace partners are underway to conduct accelerated aging tests in simulated combustor environments (e.g., 1100°C with 5% O₂ and particulate flow).

Advanced Material Integration: Exploring nanocomposite insulation layers (e.g., Al₂O₃-SiC [17]) to enhance thermal shock resistance and adhesion under cyclic loading.

Manufacturing Process Optimization: Developing hybrid deposition techniques (e.g., atomic layer deposition for protective coatings) to bridge the gap between lab-scale precision and industrial scalability.

Reviewer #2:

One of the major concerns is the lack of a strong novelty statement in the introduction (Page 2, Lines 30–55). While the introduction effectively outlines the significance of high-temperature measurements in aero engines and summarizes previous research on TFTCs, it does not clearly define what specific advancements this study contributes beyond prior works. The manuscript would benefit from an explicit statement detailing how the proposed sensor differs from existing TFTC designs, whether through material selection, fabrication techniques, or performance enhancements.

Additionally, the comparison with prior studies (Page 3, Lines 10–35) is extensive but lacks a clear gap analysis that justifies this work.

The methodology is comprehensive but lacks some critical details needed for experimental reproducibility. The preparation of the NiCrAlY transition layer (Page 5, Lines 90–110) and the Al₂O₃ insulation/protective layers (Page 6, Lines 120–135) is well described, but the manuscript does not explain why specific deposition parameters were chosen (e.g., sputtering power, target-substrate distance, annealing conditions). Given that these parameters significantly influence film adhesion, uniformity, and thermal stability, the authors should provide a clear justification or reference prior optimization studies. Furthermore, in Table 1 (Page 10, Lines 210–215), the functional layer thicknesses of PtRh and Pt films are given as 3.5 μm and 3 μm, respectively, but the rationale behind these thicknesses is not provided. A discussion on how thickness impacts sensor sensitivity and durability would enhance the study’s depth.

The discussion section should be expanded to better contextualize the practical implications of the study. While the manuscript emphasizes that the sensor can withstand extreme conditions up to 1100°C, there is little discussion on how the design could be further optimized for real-world aerospace applications. Additionally, the protective layer's role in oxidation resistance and durability (Page 6, Lines 140–150) is mentioned briefly, but it would be useful to compare this protective strategy to alternative coating methods (e.g., YSZ coatings or SiO₂ barriers) used in high-temperature sensors.

The figures and tables are well-structured, but some figure legends lack essential information. For example, Figure 3 (Page 7, Lines 160–170) presents SEM images of the NiCrAlY transition layer, but there is no scale bar or magnification information. Similarly, in Figure 5 (Page 13, Lines 290–310), the number of independent experimental replicates is not specified, which is critical for assessing reproducibility.
1. **Response:** On page 2, lines 30-55, at the end of the paragraph, add “Innovative Points of this Study”, which clearly states that: (1) For the first time, NiCrAlY alloy is used as a transition layer, and dense Al₂O₃ interfacial film is formed in-situ at the interface by vacuum oxidation to improve the high-temperature adhesion and antioxidant performance; (2) Combining electron beam evaporation and RF sputtering dual processes to prepare Al₂O₃ insulating/protective layer to realize the synergistic optimization of film density and resistance. (2) Combining the dual processes of electron beam evaporation and RF sputtering to prepare Al₂O₃ insulating/protecting layer, which achieves the synergistic optimization of film density and temperature resistance; (3) Obtaining a high Seebeck coefficient of 8.16 μV/°C and an internal error of ±1% under a combination of 3.5 μm PtRh and 3 μm Pt functional layer thickness, which significantly outperforms the existing work.

2. **Response:** On page 3, lines 10-35, a new paragraph titled "Research Gaps" is added to summarize: (1) most reports focus on single preparation processes or single material systems, neglecting the adhesion of the transition layer and the density of the protective layer; (2) few studies systematically optimize the thickness of functional layers and their high-temperature stability; (3) existing membrane couplings are mostly tested at 800-1000°C, with limited validation of high-temperature lifetimes above 1100°C. This work addresses these gaps by proposing a multi-layer collaborative optimization design and long-life verification at 1100°C.
3. **Response:** On page 5, after the description of the transition layer process in lines 90-110, add: power 100 W, target distance 60 mm: according to the literature [36,37], this parameter range makes NiCrAlY atomic kinetic energy moderate, which is conducive to Film densification and avoids particle agglomeration;

Oxidative annealing 1000 °C/vacuum + O₂: Referring to the thermal growth optimization of YSZ/Al₂O₃ composite film in Applied Surface Science 2022, this condition can generate a 50 nm dense Al₂O₃ layer in situ at the interface.

Similarly, in the Al₂O₃ insulating/protective layer section on page 6, lines 120-135, add:

E-beam evaporation combined with RF sputtering: E-beam evaporation provides a source of high-purity Al₂O₃, and RF sputtering further fills the pores and significantly improves the insulation resistance and thermal shock stability, citing the relevant optimization study by Ceramics International 2024.

**Response:** Add a thickness discussion section below Table 1:

3.5 μm/3 μm: Based on the thickness-response time and thermal resistance balance analysis of thin film couplings, the thermal response can be shortened compared with thin films, but too thin is easy to cause membrane rupture; 3-4 μm thickness is selected to take into account the improvement of Seebeck coefficient and mechanical stability, which is supported by the conclusion of thickness optimization in Review of Scientific Instruments 2021.

5. **Response:** At the end of the discussion (after lines 140-150 on the original page 6), two new sections are expanded:

Real-world application optimization: it is suggested that gradient functional layer structures or nanoporous dielectric fillings could be developed in the future for aero-engine vibration and shock environments to enhance fatigue resistance;

Comparison of protective layers: Compared with the commonly used YSZ thermal barrier coating, the Al₂O₃ protective layer has higher electrical insulation and interfacial adhesion, but slightly inferior thermal shock resistance, and it is suggested that Al₂O₃/YSZ composite coatings should be explored in subsequent work to take into account the advantages of both.

We tried our best to improve the manuscript and made some changes in the manuscript.Revised portions are marked in red in the paper. These changes will not influence the content and framework of the paper.

Once again, thank you very much for your comments and suggestions.
