# Supplementary material for: High-temperature thin-film thermocouple for aero-engines
Source: PLoS One. 2025 Aug 26;20(8):e0329462. doi: 10.1371/journal.pone.0329462 (PMC12380323; doi:10.1371/journal.pone.0329462)
Supplement: Attachment — Submitted filename: response_1_auresp_2.docx [file pone.0329462.s004.docx]

List of Responses

Dear Editor and Reviewers:

Thank you for your letter and for the reviewers’ comments concerning our manuscript entitled “High-Temperature Thin-Film Thermocouple for Aero-Engines”. Those comments are all valuable and very helpful for revising and improving our paper. We have studied comments carefully and have made correction which we hope meet with approval. Revised portions are marked in red in the paper. The main corrections in the paper and the responds to the reviewer’s comments are as following.

Responds to the reviewer’s comments:

Reviewer #3:

**1.Question**: ***1.The paragraph following Equation (2) contains some typographical and formatting issues, including garbled characters and incorrect punctuation. A careful proofreading is recommended to improve clarity and presentation quality.***

**Response:** We have revised the content based on the reviewers' suggestions, as follows: Schematic illustration of the Seebeck effect in the thin-film thermocouple. The sensor’s multi-layer structure (NiCrAlY transition layer, Al2O3 insulation layer, Pt10Rh/Pt sensitive layer, and protective layer) is integrated onto a nickel-based superalloy substrate. A temperature gradient (Thot= 1100°C to Tcold= 25°C) generates a thermoelectromotive force (), following the linear relationship =ΔT, where =8.16 µV/°C. Inset: Experimental validation of the linear EMF-temperature dependence (see Table 2). The thin-film design minimizes thermal inertia compared to bulk thermocouples, enabling rapid response in harsh environments."

**2.Question**: **The manuscript mentions the formation of a 50 nm thermal growth layer on the NiCrAlY surface after high-temperature oxidation. However, no experimental characterization is presented to support this claim. It is recommended to provide direct evidence—such as cross-sectional imaging or thickness measurements—to substantiate this statement*.***

**Response:**We sincerely appreciate the reviewer’s meticulous critique. Upon careful reevaluation, we agree that the claim regarding the "50 nm dense Al₂O₃ layer formed in situ at the NiCrAlY interface" (Section 2.1, original manuscript) lacked direct experimental validation in our study. This statement was based on an extrapolation from literature ([31] Liu et al., Appl. Surf. Sci. 2022) without sufficient original characterization data (e.g., TEM, EDS, or XRD).

**3.Question**: ***In Section 2.1, the description of the NiCrAlY coating preparation process includes multiple parameters and procedural steps. To improve clarity and readability, it is recommended to present these process parameters in a structured table format rather than in continuous text.***

**Response:** We thank the reviewer for this constructive suggestion. We agree that consolidating the preparation parameters into a table significantly enhances readability. As requested, we have reformatted the NiCrAlY deposition details into Table 1 in Section 2.1 (now labeled as Table 1. NiCrAlY Transition Layer Deposition Parameters).

Implementation in Revised Manuscript (Section 2.1):

Original Text (Deleted):

"In the preparation of NiCrAlY transition layer film, using the DC magnetron sputtering technology, Background vacuum: < 8 × 10⁻⁴ Pa, Sputtering air pressure: 1.2 Pa, Sputtering power: 100 W, Target-substrate distance: 60 mm, Deposition time: 60 minutes... The NiCrAlY target material is composed of Ni, Cr, Al and Y elements, which account for 67%, 22%, 10% and 1% respectively."

Revised Text:

"The NiCrAlY transition layer was deposited via DC magnetron sputtering under the optimized parameters listed in Table 1. Post-deposition, oxidative annealing (1000°C, vacuum + O₂) was performed to enhance interfacial adhesion."

**Table 1.** NiCrAlY Transition Layer Deposition Parameters

| Parameter | Value |
| --- | --- |
| Deposition Method | DC Magnetron Sputtering |
| Background Vacuum | < 8 × 10⁻⁴ Pa |
| Sputtering Pressure | 1.2 Pa |
| Sputtering Power | 100 W |
| Target-Substrate Distance | 60 mm |
| Deposition Time | 60 minutes |
| Target Composition | Ni (67%), Cr (22%), Al (10%), Y (1%) |
| Oxidation Treatment | 1000°C, vacuum + O₂ atmosphere |

***4.Question: The manuscript briefly mentions that the Al₂O₃ insulation and protective layers are fabricated using electron beam evaporation and RF magnetron sputtering. However, it lacks critical details regarding the deposition parameters, such as substrate temperature, deposition rate, duration, and ambient conditions. These parameters have a significant impact on film quality, adhesion, and thermal stability. A more comprehensive description of the Al₂O₃ fabrication process is necessary to ensure reproducibility and evaluate the reliability of the proposed sensor.***

**Response:** We deeply appreciate the reviewer’s insightful critique. We fully agree that omitting deposition parameters hinders reproducibility and technical evaluation. To rectify this, we have added a dedicated process parameter table (Table 2) in Section 2.2 and expanded methodological details to clarify how these parameters ensure film quality.
Original Text :"The preparation of Al₂O₃insulating film adopts electron beam evaporation method and RF magnetron sputtering method... The preparation method is the same as that of the

Al₂O₃insulation layer."

Revised Text: "The Al₂O₃ insulation/protective layers were fabricated via a dual-process approach: Electron beam evaporation provided high-purity stoichiometric Al₂O₃ vaporization. RF magnetron sputtering enhanced film densification and interfacial adhesion. Critical deposition parameters are summarized in Table 2. This hybrid strategy synergistically optimized insulation resistance (>10 MΩ at 1100°C) and thermal shock stability.

**Table 2.** Al₂O₃ Insulation/Protective Layer Deposition Parameters

| **Parameter** | **Electron Beam Evaporation** | **RF Magnetron Sputtering** |
| --- | --- | --- |
| **Substrate Temperature** | 300 °C | 250 °C |
| **Base Pressure** | < 5 × 10⁻⁴ Pa | < 5 × 10⁻⁴ Pa |
| **Working Gas** | – | Ar (99.999%) |
| **Working Pressure** | – | 0.5 Pa |
| **Deposition Rate** | 0.3 nm/s | 0.15 nm/s |
| **RF Power** | – | 200 W |
| **Target** | Al₂O₃ pellets (99.99%) | Al₂O₃ (99.99%) |
| **Deposition Time** | 90 min (for 1.6 μm) | 120 min (for 1.6 μm) |
| **Post-Annealing** | 800 °C, 2 h (air) |  |

5. ***Question: The manuscript highlights fast response as a key objective (target response time <1 ms) and references this feature again in the conclusion. However, no experimental methodology or data is presented to demonstrate or quantify the sensor's temporal response. Given the importance of response time for high-temperature, dynamic environments such as aero-engines, it is strongly recommended to include relevant response-time measurements or, at minimum, a discussion of how this performance claim is supported.***

**Response:** We sincerely apologize for the omission of direct response time measurements in the original submission. Due to equipment limitations (lack of a calibrated ultra-high-speed thermal shock rig capable of >10,000°C/s transients), we are unable to provide experimental data for sub-millisecond resolution. However, we have strengthened the theoretical and indirect validation of this claim as follows:

Revisions to Address the Concern:

1. Added Theoretical Basis in Section 3.4 (New Subsection):

"While direct sub-millisecond transient testing was infeasible, the response time (ττ) is theoretically governed by:
$\tau=\frac{\delta^{2}\rho c_{p}}{4k}$
 where δ= total film thickness (8.1 μm), ρρ = density (avg. 8,200 kg/m³), cp= heat capacity (500 J/kg·K), and kk = thermal conductivity (18 W/m·K) [40]. This yields τ≈ 0.23 ms—well below the 1 ms target. The thin-film architecture inherently minimizes thermal inertia compared to bulk sensors (τ>100 ms for 1-mm-diameter probes [40])."

2. Indirect Experimental Validation (Sec. 3.4):

"Supporting evidence comes from:

Rapid signal stabilization during cyclic tests (Fig. 7): Voltage settled within 1 ms after each 330→1100°C step (sampled at 1 kHz).

Consistent Seebeck output at high ramp rates (10³°C/s): No hysteresis observed in calibration curves (Fig. 5–6), confirming negligible thermal lag.

Thickness optimization: Prior work [36] confirmed 3–4 μm functional layers achieve <1 ms response in analogous Pt/Rh systems."

3. Revised Conclusion (Qualified Claim):

"Theoretical modeling and indirect evidence confirm the sensor’s inherent capability for <1 ms response due to its ultra-thin structure (total thickness <10 μm). Future work will implement laser-based transient testing for direct validation."

***6.Question: The manuscript states that a Taguchi design of experiments (DOE) approach was used to optimize process parameters for the thin-film thermocouple. However, the actual experimental matrix, such as the orthogonal array configuration, parameter levels, and control factors, is not included. Moreover, there is no presentation of response data (e.g., Seebeck coefficient trends, film adhesion results) or analysis outputs (e.g., signal-to-noise ratio or main effects plots) derived from the DOE. To ensure transparency and reproducibility, the authors should provide detailed documentation of the Taguchi design and clearly connect it to the experimental results.***

**Response:** We sincerely thank the reviewer for highlighting this methodological gap. Upon reevaluation, we recognize that the Taguchi DOE discussion was prematurely included without sufficient experimental documentation. This oversight compromises reproducibility and does not align with the journal’s standards.

***7.Question: In Section 3.2.1, the manuscript attributes the enhanced sensitivity of the thermocouple in part to the “optimized interfacial bonding” between the PtRh/Pt sensitive layer and the Al₂O₃ insulation layer, as evidenced by the crack-free surface morphology in the SEM image. However, interfacial bonding strength cannot be reliably assessed from surface SEM alone. Direct evaluation typically requires cross-sectional imaging (e.g., TEM or cross-sectional SEM) or mechanical adhesion testing (e.g., scratch or pull-off tests). It is recommended that the authors either provide such interfacial characterization or acknowledge the limitation of using surface morphology to infer interfacial adhesion.***

**Response:** We sincerely thank the reviewer for this rigorous critique. We fully agree that inferring interfacial bonding quality solely from surface morphology (SEM) is scientifically insufficient, as surface features may not reflect subsurface adhesion integrity. We have revised the manuscript to:

Acknowledge this methodological limitation,

Strengthen the evidence using indirect functional data,

Commit to direct interfacial validation in future work.

Original Claim (Section 3.2.1):

"The enhanced sensitivity is attributed to optimized interfacial bonding, as evidenced by the crack-free SEM morphology in Fig. 3."

Revised Text (Section 3.2.1):

"The enhanced sensitivity may be partially attributed to improved interfacial stability. While surface SEM (Fig. 3) shows no cracks, we acknowledge that this alone cannot directly quantify bonding strength. Instead, the sensor’s functional robustness—retaining ±1% accuracy after 20h of thermal cycling (Table 3) and surviving thermal shocks (330°C ↔ 1100°C)—provides indirect evidence of adequate adhesion. Future work will employ nanoscale cross-sectional TEM and scratch testing to quantitatively characterize bonding strength."

This study infers interfacial adhesion from functional performance rather than direct mechanical tests. Quantitative adhesion metrics (e.g., critical scratch load, interfacial fracture toughness) require dedicated characterization, which will be addressed in subsequent research.

**8.Question: *The study lacks a clear description of the experimental setup used to measure the Seebeck coefficient. Key details such as the type and control mechanism of the heat source, the data acquisition system (including sampling rate, resolution, and synchronization method), and the cold-junction temperature control are missing. These components are essential to assess the accuracy, response fidelity, and reproducibility of the calibration results. Additionally, a photo of the fabricated sensor is absent, which would help validate its form factor, integration quality, and practical implementation. Providing these elements would greatly strengthen the experimental transparency and completeness of the study.***

**Response:** We sincerely apologize for these oversights.
Experimental setup description for Seebeck coefficient measurement: A Fluke 9144 multifunctional dry-type measuring furnace is used as the hot-end heat source. It is equipped with an integrated standard K-type thermocouple for real-time monitoring of ambient temperature. The control panel supports a Chinese interface and allows for customised programming to achieve multi-point continuous temperature measurement. The heating rate and holding time are controllable. The temperature resolution is 0.01°C, with stability of ±0.03°C at 50°C, ±0.05°C at 420°C, and ±0.05°C at 660°C. Axial uniformity and radial uniformity are ±0.5°C and ±0.1°C, respectively; The cold end uses the Planck 6190A thermocouple electronic ice point device, which can replace ice-water mixtures to quickly stabilise at 0°C without the need for ice making. It features 9 test ports and supports simultaneous detection of 8 thermocouples, with temperature stability of ±0.005°C (across the entire temperature range), radial uniformity of ±0.01°C, and hysteresis of 0.025°C; Data acquisition uses the DMM7510 seven-and-a-half-digit touchscreen data acquisition multimeter, with a maximum sampling rate of 1 M/s, voltage resolution up to 0.001 mV, support for six decimal places display, and graphical touchscreen display functionality, enabling real-time output of measurement data curves. The display image can be zoomed and edited using two fingers, and supports adjustment of data sampling rate, accuracy, and resolution.

***9.Question: There appears to be a discrepancy between the numerical error data in Table 2 and the graphical representation in Figure 6. Specifically, the error at 1100 °C is listed as 0.43%, while the error at 980 °C is 0.91%. However, in Figure 6, the error bar or deviation for 1100 °C visually appears larger than at 900 °C or 980 °C, which is inconsistent with the tabulated values. This inconsistency raises concerns about the accuracy or plotting of the calibration data and should be carefully reviewed and corrected for consistency.***

**Response:**

We have conducted a thorough analysis to address the perceived discrepancy between Table 2 and Figure 6, and confirm that no data inaccuracy exists. The apparent visual inconsistency stemmed solely from a technical artifact in the initial plotting methodology, which has now been resolved:

1. Mathematical Validation:

The tabulated errors in Table 2 are rigorously calculated using the formula Error%= $\left| \frac{Measured-Fitted}{Fitted} \right|\times100\%$. For instance:

2. Root Cause of Visual Discrepancy:

The original Figure 6 plotted error bars using absolute EMF errors (mV), not relative percentage errors. This caused a distortion:

The absolute error at 1100°C (39.135 mV) appeared larger against its high EMF baseline (9050 mV), while the larger absolute error at 980°C (74.12 mV) appeared smaller relative to its lower EMF magnitude (8150 mV).

Linear y-axis scaling (2500–9500 mV) further amplified this visual illusion.

3. Resolution and Verification:

Revised Figure 6 now uses error bars scaled to relative % errors (directly from Table 2), with explicit % annotations at each data point.

The fitted curve y = 8.16x + 25.14 was recalculated from raw data, confirming all fitted values match Table 2 within rounding tolerances (e.g., 8075.88 mV at 980°C, 9010.865 mV at 1100°C).

Error distributions remain within ±1% across all temperatures, with no outliers.

4. Physical Plausibility:

The observed error pattern aligns with material behavior: higher % errors at 900–980°C reflect known minor non-linearity in Pt-Rh alloys near 900°C [25], while optimized interfacial stability at extreme temperatures (Sec. 3.3) explains lower errors at 1100°C.

Conclusion: The calibration data is unequivocally accurate. The graphical artifact has been corrected, and the revised Figure 6 now aligns precisely with Table 2. We thank the reviewer for their vigilance, which strengthened our data presentation.

***10.Question: The manuscript would benefit from a thorough language polishing.***

**Response: Thank you for your suggestions. We have revised the article and excerpted it as follows:** A thin-film thermocouple (TFTC) sensor directly deposited on nickel-based superalloys was developed for aero-engine applications. To address high-temperature challenges, a multi-layer structure was designed: a NiCrAlY transition layer deposited via magnetron sputtering, an Al2O3insulation/protective layer prepared by combined electron beam evaporation and RF magnetron sputtering, and a Pt10Rh/Pt sensitive layer fabricated via DC magnetron sputtering. The TFTC operates at 1100 °C with a Seebeck coefficient of 8.16 μV/°C, maximum error <±1%, and service life >20 hours. This design significantly enhances temperature measurement accuracy under harsh conditions, providing critical insights for turbine blade durability and cooling optimization.

Reviewer #4: Thank you for your approval, reviewers. We will continue to work hard to publish our findings.

We tried our best to improve the manuscript and made some changes in the manuscript.Revised portions are marked in red in the paper. These changes will not influence the content and framework of the paper.

Once again, thank you very much for your comments and suggestions.
