# Supplementary material for: High-temperature thin-film thermocouple for aero-engines
Source: PLoS One. 2025 Aug 26;20(8):e0329462. doi: 10.1371/journal.pone.0329462 (PMC12380323; doi:10.1371/journal.pone.0329462)
Supplement: Attachment — Submitted filename: Reviewer Comments for Manuscript.pdf [file pone.0329462.s001.pdf]

Development and Performance Research of Nickel-Based Alloy Thin-Film Thermocouple Sensor for Aero Engine

Decision: Major revision

Detailed Comments to the Authors

The manuscript "Development and Performance Research of Nickel-Based Alloy Thin-Film Thermocouple Sensor for Aero Engine" presents a study on the design, fabrication, and performance evaluation of a thin-film thermocouple (TFTC) directly deposited on nickel-based superalloy substrates for high-temperature aerospace applications. The study is well-structured and provides a clear experimental approach, but several areas require improvement to enhance scientific rigor, reproducibility, and clarity.

One of the major concerns is the lack of a strong novelty statement in the introduction (Page 2, Lines 30–55). While the introduction effectively outlines the significance of high-temperature measurements in aero engines and summarizes previous research on TFTCs, it does not clearly define what specific advancements this study contributes beyond prior works. The manuscript would benefit from an explicit statement detailing how the proposed sensor differs from existing TFTC designs, whether through material selection, fabrication techniques, or performance enhancements. Additionally, the comparison with prior studies (Page 3, Lines 10–35) is extensive but lacks a clear gap analysis that justifies this work.

The methodology is comprehensive but lacks some critical details needed for experimental reproducibility. The preparation of the NiCrAlY transition layer (Page 5, Lines 90–110) and the Al_2O_3 insulation/protective layers (Page 6, Lines 120–135) is well described, but the manuscript does not explain why specific deposition parameters were chosen (e.g., sputtering power, target-substrate distance, annealing conditions). Given that these parameters significantly influence film adhesion, uniformity, and thermal stability, the authors should provide a clear justification or reference prior optimization studies. Furthermore, in Table 1 (Page 10, Lines 210–215), the functional layer thicknesses of PtRh and Pt films are given as 3.5 μm and 3 μm, respectively, but the rationale behind these thicknesses is not provided. A discussion on how thickness impacts sensor sensitivity and durability would enhance the study’s depth.

The discussion section should be expanded to better contextualize the practical implications of the study. While the manuscript emphasizes that the sensor can withstand extreme conditions up to 1100°C, there is little discussion on how the design could be further optimized for real-world aerospace applications. Additionally, the protective layer's role in oxidation resistance and durability (Page 6, Lines 140–150) is mentioned briefly, but it would be useful to compare this protective strategy to alternative coating methods (e.g., YSZ coatings or SiO_2 barriers) used in high-temperature sensors.

The figures and tables are well-structured, but some figure legends lack essential information. For example, Figure 3 (Page 7, Lines 160–170) presents SEM images of the NiCrAlY transition layer, but there is no scale bar or magnification information. Similarly, in Figure 5 (Page 13, Lines 290–

310), the number of independent experimental replicates is not specified, which is critical for assessing reproducibility.
